# On‐Surface Synthesis and Characterization of Cumulene‐Linked Stone‐Wales Polymers

**DOI:** 10.1002/anie.202506803

**Published:** 2025-07-25

**Authors:** Elena Pérez‐Elvira, Fupeng Wu, Jeong Ha Hwang, Ji Ma, Lucia Palomino‐Ruiz, Sofia Canola, Ana Barragán, Koen Lauwaet, José M. Gallego, Rodolfo Miranda, Mickael L. Perrin, David Écija, Aurelio Gallardo, Gabriela Borin Barin, Xinliang Feng, José I. Urgel

**Affiliations:** ^1^ IMDEA Nanoscience C/ Faraday 9, Campus de Cantoblanco Madrid 28049 Spain; ^2^ Max Planck Institute of Microstructure Physics Weinberg 2 06120 Halle Germany; ^3^ Empa‐Swiss Federal Laboratories for Materials Science and Technology Überlandstrasse 129 Dübendorf 8600 Switzerland; ^4^ Department of Information Technology and Electrical Engineering ETH Zurich Zurich 8092 Switzerland; ^5^ College of Materials Science and Optoelectronic Technology &Center of Materials Science and Optoelectronics Engineering University of Chinese Academy of Science Beijing 100049 P. R. China; ^6^ Departamento de Química Orgánica Facultad de Ciencias, Universidad de Granada, Unidad de Excelencia en Química (UEQ) Granada 18071 Spain; ^7^ Institute of Physics of the Czech Academy of Science Cukrovarnická 10 Praha 162 00 Czech Republic; ^8^ Instituto de Ciencia de Materiales de Madrid (ICMM), CSIC Cantoblanco Madrid 28049 Spain; ^9^ Quantum Center, ETH Zürich Zürich 8093 Switzerland; ^10^ Unidad de Nanomateriales avanzados, Imdea Nanoscience, Unidad asociada al CSIC por el ICMM Madrid 28049 Spain; ^11^ Center for Advancing Electronics Dresden (cfaed) & Faculty of Chemistry and Food Chemistry Technische Universität Dresden D‐01069 Dresden Germany

**Keywords:** Carbon‐based polymers, Non‐contact atomic force microscopy, Scanning tunneling microscopy, Stone‐Wales defect, Surface chemistry

## Abstract

Structural, chemical, and extrinsic modifications of graphene‐based nanostructures enable bandgap tuning, optoelectronics, spintronics, and quantum materials design. A well‐known approach to modify their electronic properties involves introducing nonbenzenoid ring topologies in their ideal sp^2^‐hybridized hexagonal lattice, such as azulene or Stone‐Wales (SW) defects. However, despite the unique structural and electronic characteristics that these nonalternant defects induce, their systematic incorporation in graphene‐based nanostructures remains challenging. Here, we demonstrate the on‐surface synthesis of one‐dimensional SW‐based polymers linked through cumulene bonds on the Au(111) surface via thermal and visible‐light‐induced reactions of a tailored molecular precursor. Scanning tunneling and noncontact atomic force microscopies reveal the nonplanar structure of SW‐based units within the polymer chain, while the chemical structure of the polymer has been verified by Raman spectroscopy in combination with theoretical modeling. Additionally, scanning tunneling spectroscopy measurements show an experimental bandgap of 1.8 eV, which significantly differs from its isostructural cumulene‐bridged bisanthene analogs. Our results highlight the critical role of SW defects in the structural and electronic properties of carbon‐based conjugated polymers, advancing their design with prospects in next‐generation optoelectronic devices.

## Graphical abstract

On‐surface synthesis of one‐dimensional polymers featuring Stone‐Wales defects and cumulene linkages is demonstrated via thermally and light‐induced reactions on Au(111). STM, nc‐AFM, and Raman spectroscopy, complemented by theory calculations, reveal their chemical structure with a non‐planar geometry, and a distinct electronic bandgap of 1.8 eV, underscoring the impact of nonbenzenoid motifs in tuning the properties of carbon‐based nanomaterials.

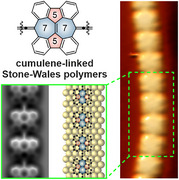

The exceptional physicochemical properties of graphene paved the way for groundbreaking progress in materials science and technology.^[^ ^1^, ^2^ ^]^ However, its integration into the optoelectronic industry remains constrained by the zero bandgap of its ideal sp^2^‐hybridized hexagonal lattice. In recent decades, various structural, chemical and external modification strategies have facilitated the tuning of the electronic properties in graphene‐based nanostructures. For instance, the on‐surface fabrication of one‐dimensional (1D) graphene stripes, i.e., graphene nanoribbons, with precise widths and edge configurations has proven to be promising,^[^ ^3^, ^4^ ^]^ as quantum confinement in these nanostructures induces the opening of bandgaps, positioning them as potential candidates for nanoscale semiconductors and next‐generation optoelectronic devices.^[^ ^5^, ^6^, ^7^ ^]^

Another pathway to tailoring the electronic properties of these graphene nanostructures involves introducing controlled imperfections or defects in their basal honeycomb lattice. Common defects include atom dislocations and substitutions, point defects, vacancies, adatoms, and edge reconstructions, all of which have been both theoretically predicted and experimentally observed.^[^ ^8^ ^]^ Among them, the incorporation of non‐hexagonal ring topologies,^[^ ^9^ ^]^ like the Stone‐Thrower‐Wales point defect, also abbreviated as Stone‐Wales (SW) defect, is unique in terms of their structural, electronic and quantum properties.^[^ ^10^ ^]^ The SW defect is formed through a 90° rotation of a carbon–carbon bond in two adjacent hexagons, resulting in a pair of pentagonal and heptagonal rings. This defect has been directly observed via transmission electron microscopy (TEM)^[^ ^11^ ^]^ and, more recently, scanning tunneling microscopy (STM),^[^ ^12^, ^13^ ^]^ although mainly focusing on the structural characterization. Although the physicochemical properties of isolated SW defects have been extensively investigated, their controlled and systematic incorporation into extended graphene‐based nanostructures, and the resulting influence on structure‐property relationship remain challenging, which motivates a thorough understanding both from fundamental and applied perspectives. In this regard, surface‐assisted techniques under ultra‐high vacuum (UHV) conditions have emerged as a powerful approach toward the design and characterization of carbon‐based nanostructures that were previously challenging to produce, effectively overcoming limitations associated with conventional synthetic methods.^[^ ^14^ ^]^

In this communication, we present the first example of a 1D polymer incorporating SW defects connected through cumulene linkages, synthesized directly on a metal surface. The polymers result from the thermally and visible‐light‐induced on‐surface reactions of the molecular precursor 7,14‐bis(dibromomethylene)‐7,14‐dihydroheptaleno[2,1,10,9‐*jklm*:4,5,6,7‐*j'k'l“m”*]difluorene (**1**) on a bare Au(111) surface (Figure 1a). Structural characterization by STM and non‐contact atomic force microscopy (nc‐AFM), complemented by Raman spectroscopy, reveals a distinct non‐planar geometry of the SW units within the polymer chain. Importantly, the dual activation strategy, thermal and visible light, adds versatility to the synthetic protocol. Furthermore, scanning tunneling spectroscopy (STS), supported by theoretical calculations, reveals an experimental bandgap of 1.8 eV. Notably, significant structural and electronic differences arise when compared to its isostructural cumulene‐bridged bisanthene analogs fabricated on the same substrate,^[^ ^15^ ^]^ highlighting how SW defects can be systematically integrated to tune the electronic properties of graphene‐based nanostructures.

**Figure 1:**
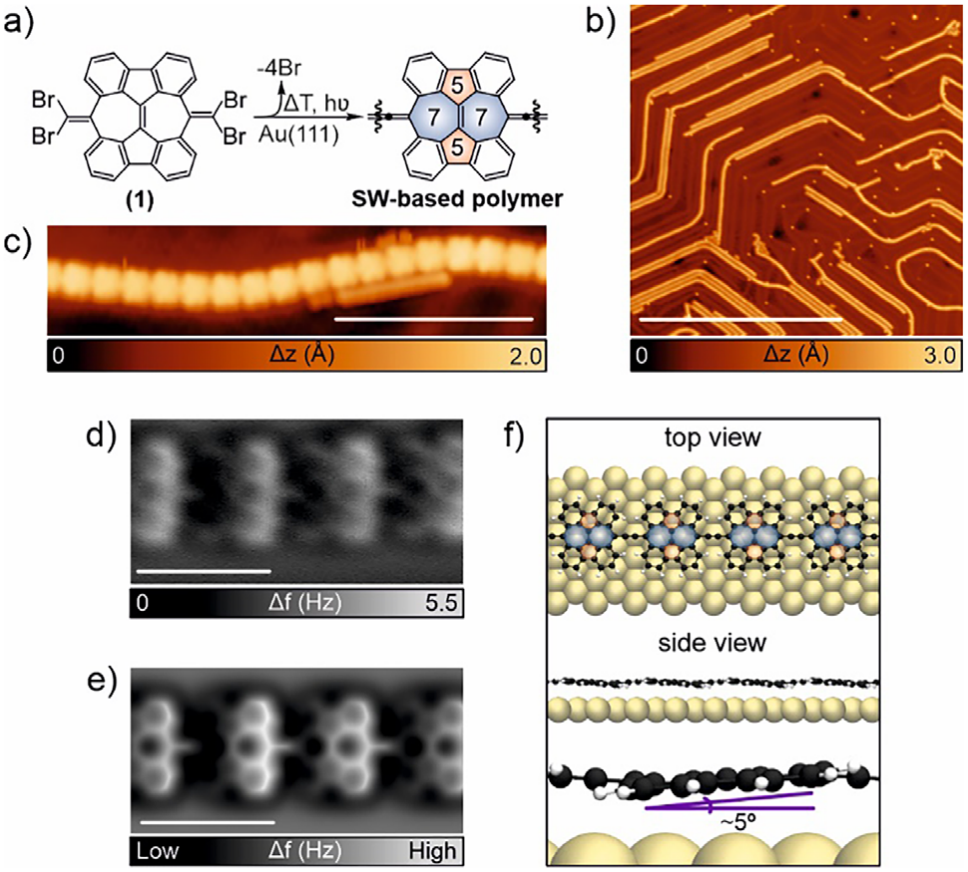
Structural characterization of cumulene‐linked SW polymers synthesized on Au(111). a) General on‐surface synthesis sketch of the polymer reported in this work. The SW defects are highlighted in blue (seven‐membered rings) and red (five‐membered rings). b) Large‐scale and c) high‐resolution STM images of the obtained polymers. Scanning parameters: (b) *V*_b_ = 0.2 V, *I*_t_ = 30 pA and scale bar = 50 nm; (c) *V*_b_ = 0.5 V, *I*_t_ = 30 pA and scale bar = 5 nm. d) Constant‐height frequency‐shift nc‐AFM image of a polymer segment depicted in panel (c) acquired with a CO‐functionalized tip (z‐offset 120 pm below STM set point: 5 mV, 50 pA). e) Simulated nc‐AFM image of panel (d). f) Top and side views of the DFT equilibrium geometry of the polymer backbone on Au(111), together with the angle of a SW‐based unit.

The synthesis of precursor **1** was accomplished in solution via a six‐step procedure (Scheme 1), starting from commercially available fluoranthene (**2**). First, the oxidation of **2** with chromium trioxide yielded 9‐fluorenone‐1‐carboxylic acid (**3**) in 70% yield. The carboxyl group in compound **3** was then esterified, affording methyl 9‐fluorenone‐1‐carboxylate (**4**) in 88% yield. Next, tosylhydrazone derivative **5** was prepared from compound **4** in 90% yield. Treatment of **5** with sodium methoxide in dry pyridine generated a diazofluorene intermediate, which was immediately decomposed in the presence of CuBr, yielding dimethyl‐[9,9′‐bifluorenylidene]‐1,1′‐dicarboxylate as a mixture of (*E*)‐ and (*Z*)‐isomers in 90% yield over two steps. The cyclization reaction of **6** by heating with trifluoromethanesulfonic acid quantitatively yielded dione **7**, featuring two fused pentagon‐heptagon units. Finally, the key precursor, 7,14‐bis(dibromomethylene)‐7,14‐dihydroheptaleno[2,1,10,9‐*jklm*:4,5,6,7‐*j'k'l“m”*]difluorene (**1**) was synthesized from **7** in 37% yield via the first step of the Corey–Fuchs reaction. The chemical structure of **1** containing a SW unit was unambiguously confirmed by high‐resolution mass spectrometry, nuclear magnetic resonance (NMR) spectroscopy, and single‐crystal X‐ray diffraction (See Supporting Information and the inset in Scheme 1). To fabricate the SW‐based polymers, precursor **1** was first sublimed under UHV conditions onto an atomically clean Au(111) surface held at room temperature (see Figure S1 for the three considered absorption configurations) and subsequently annealed at 200 °C to induce debromination and homocoupling (Figure 1a). The use of molecular precursors functionalized with dibromomethylenes has been recently demonstrated as a successful strategy toward fabricating cumulene/ethynylene‐bridged polymers on surfaces.^[^ ^16^ ^]^ Figure 1b shows an overview STM image taken after the annealing treatment, revealing the presence of chain‐like nanostructures that predominantly follow the FCC regions of the underlying Au(111) herringbone reconstruction. A high‐resolution STM image of a prototype polymer displays how they are constituted of linked rectangular shape nanostructures (Figure 1c). To get further insights about their chemical structures, we perform nc‐AFM measurements using a carbon monoxide (CO)‐functionalized tip.^[^ ^17^ ^]^ Figure 1d depicts a constant‐height frequency‐shift image where each of the rectangular shape nanostructures, described in Figure 1c, shows an increased frequency shift of their right edges, in comparison to the ones on the left. We attribute this variation of the frequency shift to the nonplanar geometry of each SW‐based unit within the polymer. Furthermore, nc‐AFM images resolve a sharp line with a homogeneous contrast in the connections between SW‐based units, which is an indication of the cumulene‐like nature of the bond,^[^ ^15^, ^18^, ^19^, ^20^ ^]^ in contrast to ethynylene‐like bonds, which exhibit an enhanced contrast at their central positions (see Figure S2 for nc‐AFM images acquired at different tip heights).^[^ ^15^, ^21^, ^22^, ^23^, ^24^, ^25^, ^26^, ^27^ ^]^ Our structural assumptions are well reproduced by the simulated nc‐AFM image depicted in Figure 1e. Further annealing of the sample at 250 °C gave rise to a two‐fold cyclization reaction between adjacent SW‐based units^[^ ^28^ ^]^ without finding any hint of isomerization of the SW‐based cumulene‐bridged chains (see Figure S3). In addition, the DFT‐optimized structure of the polymer used to obtain the simulated nc‐AFM image (Figure 1f) on Au(111) suggests a tilted adsorption dihedral angle of the SW‐based unit within the polymer backbone of approximately 5°, being the minimum and maximum carbon adsorption height of 3.2 and 3.8 Å, respectively, and the carbons in the cumulene bonds of 3.4–3.7 Å, with respect to the gold underlying surface. We attribute this slight tilt from steric hindrance between hydrogen atoms of adjacent molecular units, which prevents the polymer from adopting a fully planar geometry (see Figure S4). The chain‐surface interaction energy (−2.89 eV per molecule) and the lack of strong electronic density redistributions upon absorption suggest the rather physisorption of the chains onto the surface (See Figure S5 for the adsorption height of all the carbon atoms of a finite 1D SW‐based oligomer with respect to the gold surface and the charge redistribution due to the chain‐surface interaction). This contrasts other works realized on Cu(111),^[^ ^29^ ^]^ where enhanced charge transfer of azupyrene units was reported to be stronger than in the present case. Such a difference could arise from the fact that the SW‐based polymers are tilted by the internal conformation of the polymer, affecting the sample‐substrate interaction, and the different metallic substrate employed in our work.

**Scheme 1:**
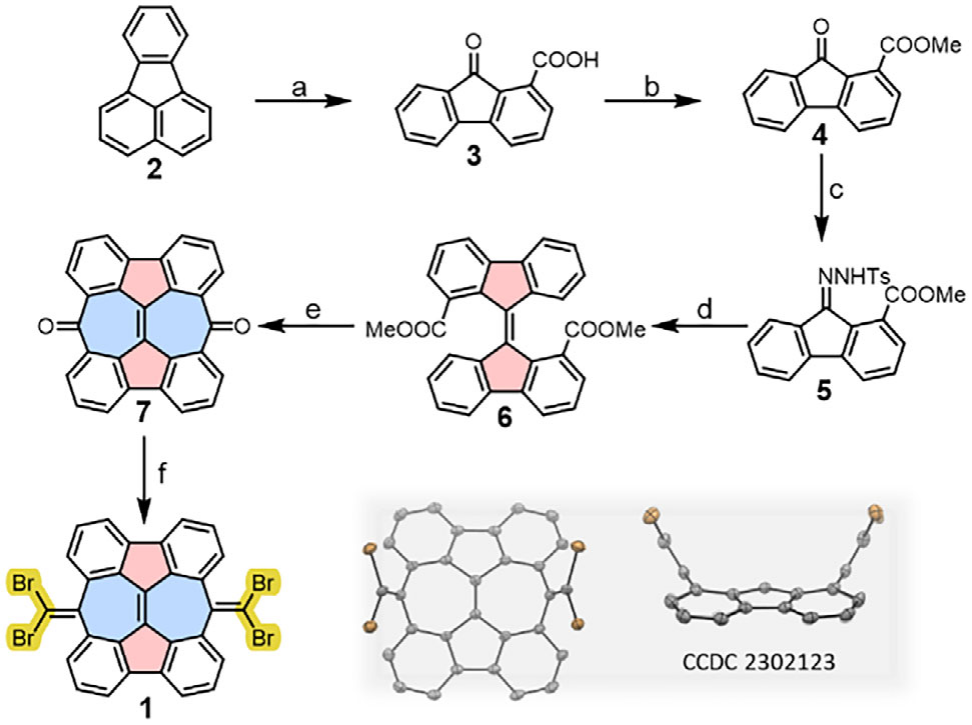
Synthetic route to precursor **1**. Reagents and conditions: a) CrO_3_, CH_3_COOH/H_2_O, 100 °C, 2 h, 70%. b) MeOH, H_2_SO_4_, 80 °C, 20 h, 88%. c) NH_2_NHTs, HCl, CH(OCH_3_)_3_, CH_3_OH, 75 °C, 16 h, 90%. d) i. NaOCH_3_, pyridine, 110 °C, 6 min; ii. CuBr, CH_2_Cl_2_, reflux, 15 min, 90% yield over two steps. e) TfOH, 90 °C, 3 h, quantitative yield. f) CBr_4_, PPh_3_, toluene, reflux, 12 h, 37%.

The inspection of the polymer terminations gives valuable information about its topological quantum phase^[^ ^15^ ^]^ and its chemical reactivity. The above‐mentioned polymers, obtained through a thermal annealing treatment, present irregular terminations (statistics out of 20 polymer terminations), as observed in the STM or nc‐AFM images, including the entire loss of dibromomethylene functional groups and passivation by residual atomic hydrogen, formation of nonbenzenoid rings or strong interaction of the terminal carbon atom with the gold surface (see Figure S6). Consequently, we have explored the use of a different activation stimulus to induce the formation of the SW‐based polymers. Light, like temperature, is also able to trigger on‐surface chemical reactions.^[^ ^30^, ^31^ ^]^ In fact, several investigations of photochemical reactions using UV/visible‐light‐induced on semiconducting or insulating^[^ ^32^, ^33^, ^34^, ^35^, ^36^, ^37^, ^38^, ^39^ ^]^ and single‐crystalline metal substrates^[^ ^40^, ^41^, ^42^, ^43^, ^44^, ^45^, ^46^, ^47^ ^]^ are reported in literature.

Therefore, we irradiate our sample, containing a submonolayer coverage of **1,** with a visible light LED (λ = 470 nm) for 15 h (see details about the illumination parameters and sample temperature after light irradiation in Supporting Information methods and Figure S7). Figure 2a shows a large‐scale STM image, where the successful formation of polymers and oligomers upon light irradiation is manifested. A closer inspection of these nanostructures reveals that they are composed of rectangular shape units with a bright protrusion at each of their edges (Figure 2b, statistics out of 20 polymer terminations). Figure 2c displays a constant‐height frequency‐shift image of an oligomer constituted of four SW‐based units, clearly showing two features of increased frequency shift at both oligomer edges. We attribute these features to the presence of intact dibromomethylene groups at the oligomer termination, as reproduced by the simulated nc‐AFM image and chemical sketch depicted in Figure 2d.

**Figure 2:**
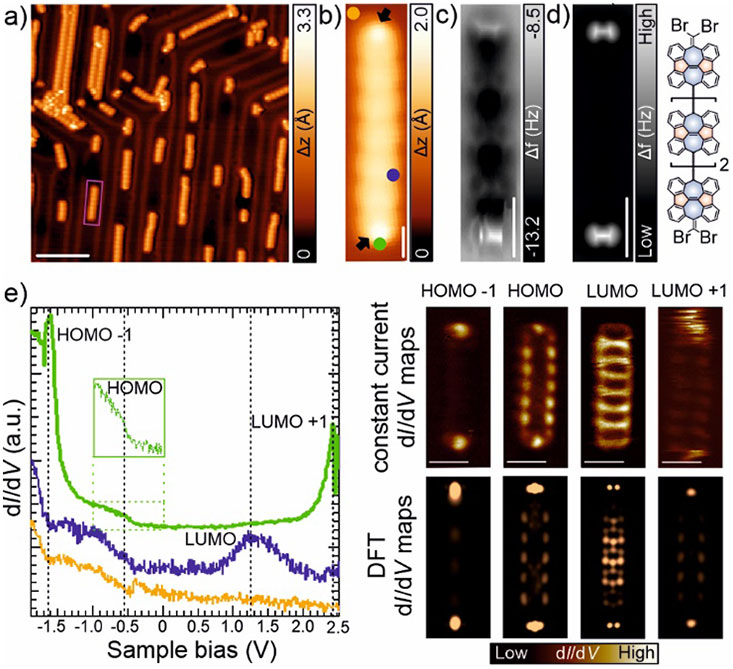
Light‐induced formation of the cumulene‐linked SW polymers together with their electronic characterization. a), b) Large‐scale and high‐resolution STM images of the sample after light illumination. Scanning parameters: (a) *V*_b_ = 0.5 V, *I*_t_ = 50 pA and scale bar = 10 nm; (b) *V*_b_ = −2.0 V, *I*_t_ = 50 pA and scale bar = 2 nm. The pink rectangle in (a) highlights the SW‐polymer shown in (b). c) Constant‐height frequency‐shift nc‐AFM image of an oligomer acquired with a CO‐functionalized tip (z‐offset of 170 pm below STM set point: 5 mV, 20 pA, scale bar = 1 nm). d) Simulated nc‐AFM image of panel (c), together with the chemical sketch of the oligomer showing the dibromomethylene terminations. Scale bar = 1 nm e) Left panel: d*I*/d*V* spectrum acquired on the oligomer shown in panel (b) at the positions indicated by the blue and green circles in such panel, revealing a HOMO‐LUMO gap of 1.8 eV. The reference spectrum was taken on the bare Au(111) surface (orange circle). Open feedback parameters: V_b_ = 2.5 V, I_t_ = 250 pA and V_rms _= 10 mV. Right panel: Constant‐current differential conductance d*I*/d*V* maps (top) and corresponding DFT‐calculated LDOS maps (bottom) at the frontier orbitals of the oligomer. Tunneling parameters for the d*I*/d*V* maps: HOMO‐1 (V*_b_* = −1.60 V, I*_t _* = 350 pA); HOMO (V*_b_* = ‐0.55 V, I*_t_* = 350 pA); LUMO (V*_b_* = 1.25 V, I*_t_* = 350 pA) and LUMO + 1 (V*_b_* = 2.40 V, I*_t_* = 350 pA).

To have an indication of the light‐mediated excitation mechanism, we computed the optical absorption spectrum of the isolated precursor **1** (Figure S8a). The calculation suggests the maximum of the lowest energy band around 394 nm (Figure S8b and Table S1). Also, considering a possible overestimation of the computed excitation energy and the peak broadening resulting from the interaction with the metal surface,^[^ ^48^ ^]^ the molecular optical absorption can be expected to be centered at an energy higher than that of the exciting laser. Thus, we speculate that the debromination light‐driven reaction is dominantly initiated by an indirect process,^[^ ^42^ ^]^ where photons are absorbed by the surface and produce hot charge carriers which enter the unoccupied states of the molecules via an inelastic scattering process, inducing the debromination and subsequent polymerization. A more detailed mechanistic analysis is beyond the scope of this work and will be the focus of future investigations.

Next, we perform STS measurements in order to probe the electronic structure. Differential conductance d*I*/d*V* spectra show features that derive from the HOMO‐1, HOMO, LUMO, and LUMO+1 of the oligomer shown in Figure 2b, being experimentally detected at −1.70, −0.55, 1.25, and 2.40 eV, respectively (Figure 2e, left panel). Simulated d*I*/d*V* maps using DFT‐calculated frontier molecular orbitals with a CO‐tip of the free‐standing oligomer match well the experimental results (Figure 2e, right panel). Therefore, the HOMO–LUMO gap of these oligomer on Au(111) is found to be 1.8 eV. This value remains consistent regardless of the polymer length and activation pathway (thermal (Figure S9) or photo‐induced (Figure 2e)). Importantly, the absence of edge states within the gap indicates a trivial insulator quantum classification for the SW polymers featuring a = CBr_2_ termination, in contrast to the topologically non‐trivial isostructural bisanthene cumulene‐bridged one displaying a ‐CH terminus.^[^ ^15^ ^]^ Comparing the studied SW‐based polymer with previously reported isostructural cumulene‐bridged bisanthene polymers shed striking structural and electronic differences (see Figure S10 for structure comparison).^[^ ^15^ ^]^ Bisanthene polymers are adsorbed planar on the Au(111) surface with a reported narrow bandgap of ∼0.3 eV, however, SW‐based polymers adopt a non‐planar or tilted geometry upon adsorption due to the presence of nonbenzenoid rings, leading to a significant increase in the electronic bandgap to ∼1.5 eV, despite both being linked through cumulene bonds. Such differences highlight the influence of the SW defects in the structural and electronic properties of graphene‐based nanostructures.

Finally, we used Raman spectroscopy, which provides a surface‐averaged view of material quality across areas spanning tens of micrometers,^[^ ^49^ ^]^ while maintaining sensitivity to atomic‐scale structural details, to further characterize the polymer structure, identifying the characteristic vibrational modes. This method is highly effective for investigating carbon nanostructures, offering insights into chirality,^[^ ^50^ ^]^ width,^[^ ^51^ ^]^ and length.^[^ ^52^ ^]^ The Raman spectra of a high coverage sample (Figure S11a), measured in the 10^−7^ mbar range and upon exposure to air (two hours and thirty minutes) are shown in Figure 3a (see Methods for measurements details). We observe a rich spectrum with many peaks between ∼1000–2000 cm^−1^. The sample measured in the 10^−7^ mbar range shows peaks at high frequencies at ∼1034, 1169, 1197, 1296, 1459, and 1545 cm^−1^, which we identify as different C─H bending modes at the edges of the polymer, as indicated by the normal mode analysis extracted from the gas phase DFT simulations on a finite‐sized SW polymer composed of four monomers using the ORCA 5.0 DFT code, PBE exchange‐correlation functional, and def2‐SVP basis set. (Figure S11b). At even higher frequencies, we observe two peaks at 1575 and 1626 cm^−1^. Compared to the DFT simulations, where peaks are centered at 1600 and 1631cm^−1^, we conclude that those are due to the C─C stretching vibrational modes.

**Figure 3:**
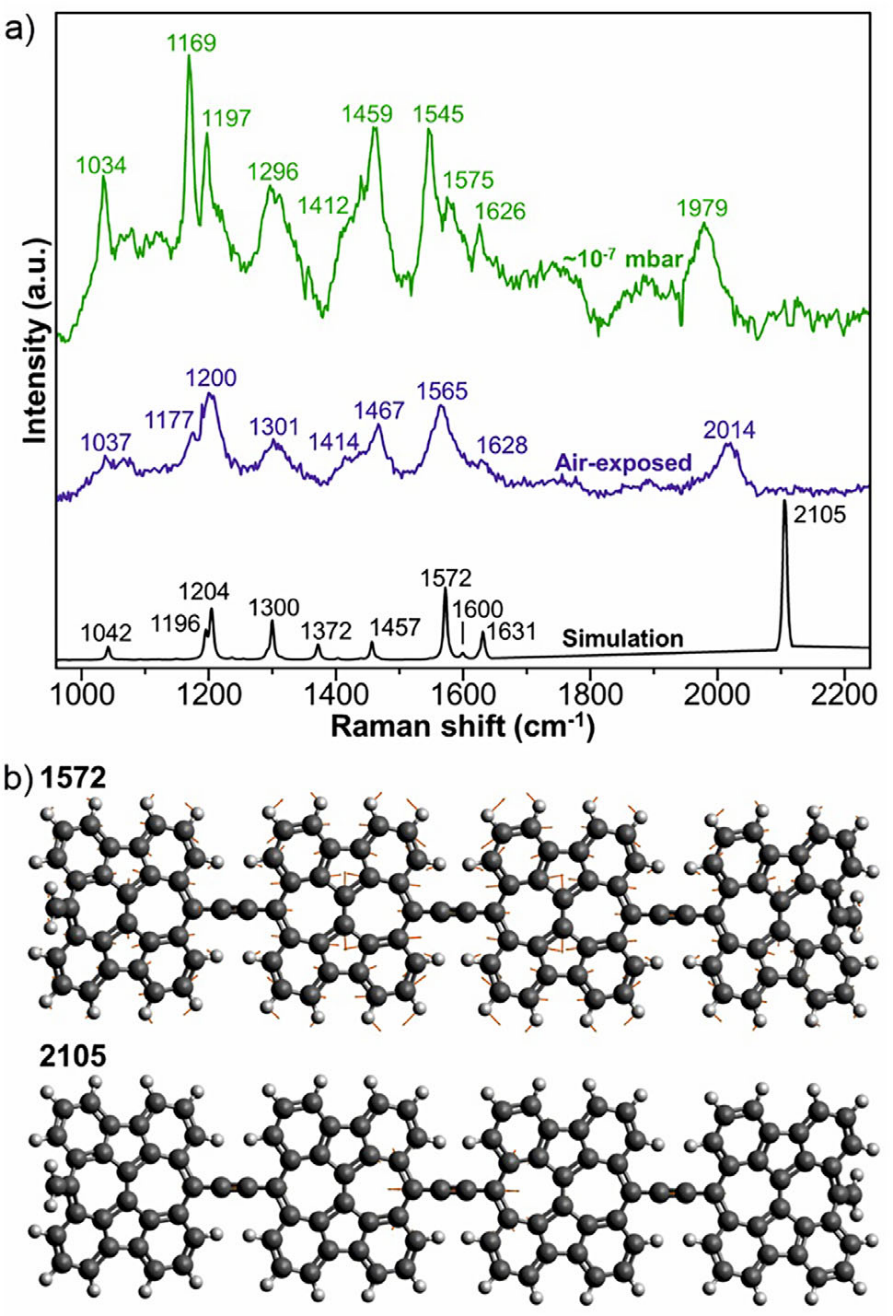
Experimental and DFT‐simulated Raman spectra of SW polymers. a) Raman spectra of the SW‐polymers collected experimentally in the 10^−7^ mbar range (green), after air exposure (blue), and by DFT simulation (black). b) Normal mode analysis of the polymer in the high‐frequency range of ∼1570–2100 cm^−1^. Red arrows indicate direction and magnitude of the atomic displacements.

The vibrational frequency of the C═C stretching mode in cumulenes is typically observed in the range of 1800–2000 cm^−1^.^[^ ^53^ ^]^ Here, we observe a peak at 1979 cm^−1^ in the sample measured in the 10^−7^ mbar range and at 2014 cm^−1^ after air exposure. Based on our Raman simulations (Figure 3a, black spectrum) and corresponding normal mode analysis (Figures 3b and S11), we attribute those modes to the cumulene bonds between the SW‐polymer (as corroborated by the normal mode analysis shown in Figure 3b).^[^ ^54^ ^]^ In shorter cumulene chains, the C═C bonds are closer to ideal double bonds, leading to higher stretching frequencies, e.g., near 2000 cm^−1^, as observed in this present study. Note that the absolute value of the higher frequency modes shows larger shifts in the DFT calculation compared to the experiment. This is a signature of DFT's inability to include many‐body effects, including those related to strong electron‐phonon coupling.^[^ ^55^ ^]^ After exposure to air, many of the modes are still present, albeit with reduced intensity and slightly shifted frequencies. Notably, the mode at ∼2000 cm^−1^, which we attribute to the cumulene bond, is preserved. This suggests that the polymer does not undergo significant structural modification after air exposure, a crucial property for the device integration of such 1D nanostructures.

In summary, our study introduces a versatile approach toward the fabrication of 1D cumulene‐linked SW‐based polymers on a single‐crystalline Au(111) surface in an UHV environment. Two polymerization strategies—thermal annealing and visible‐light activation of a dibromomethylene‐functionalized precursor—are shown to produce identical polymer backbones but different terminations, with irregular ends resulting from annealing and dibromomethylene terminations from light activation. The detailed chemical structure of the obtained polymers has been unambiguously unveiled by STM and nc‐AFM showing the non‐planar geometry of the SW‐based units, as well as Raman spectroscopy. Additionally, STS measurements, together with theoretical calculations, manifest that the cumulene‐linked SW‐based polymers exhibit a substantially larger bandgap (1.8 eV) compared with the isostructural cumulene‐bridged bisanthene polymers (0.3 eV). Finally, the absence of edge states within the gap indicates a trivial insulator quantum classification for the SW polymers featuring a = CBr₂ termination, in contrast to the topologically non‐trivial isostructural bisanthene cumulene‐bridged analogue displaying a –CH terminus. This work underscores the influence of SW defects on the structural and electronic properties of carbon‐based polymers, advancing the design of materials with tailored band structures and topological character for next‐generation optoelectronic and quantum devices. Moreover, it opens up exciting opportunities for engineering the topological properties of carbon‐based nanostructures through controlled incorporation of nonbenzenoid motifs and edge terminations.

## Conflict of Interests

The authors declare no conflict of interest.

## Supporting information



Supporting information

## Data Availability

The data that support the findings of this study are available from the corresponding author upon reasonable request.
