# Supplementary material for: On‐Surface Synthesis and Characterization of Cumulene‐Linked Stone‐Wales Polymers
Source: Angew Chem Int Ed Engl. 2025 Jul 25;64(37):e202506803. doi: 10.1002/anie.202506803 (PMC12416461; doi:10.1002/anie.202506803)
Supplement: Supplementary file 1 — Supporting information [file ANIE-64-e202506803-s001.docx]

Supporting Information
©Wiley-VCH 2021
69451 Weinheim, Germany

**On-Surface Synthesis and Characterization of Cumulene-Linked Stone-Wales Polymers**

Elena Pérez-Elvira,^+[a]^ Fupeng Wu,^+[b]^ Jeong Ha Hwang,^[c,d]^ Ji Ma,^*[b,e]^ Lucia Palomino-Ruiz,^[c,f]^ Sofia Canola,^[g]^ Ana Barragán,^[a]^ Koen Lauwaet,^[a]^ José M. Gallego,^[h]^ Rodolfo Miranda,^[a]^ Mickael L. Perrin,^[c,d,i]^ David Écija,^[a,j]^ Aurelio Gallardo,^*[a]^ Gabriela Borin Barin,^*[c]^ Xinliang Feng,^[b,k]^ and José I. Urgel^*[a,j]^

[a] Elena Pérez-Elvira, Dr. Ana Barragán, Dr. Koen Lauwaet, Prof. Rodolfo Miranda, Prof. David Écija, Dr. Aurelio Gallardo and Dr. José I. Urgel

IMDEA Nanoscience, C/ Faraday 9, Campus de Cantoblanco, 28049 Madrid, Spain
E-mail: jose-ignacio.urgel@imdea.org

[b] Dr. Fupeng Wu, Dr. Ji Ma and Prof. Xinliang Feng

Max Planck Institute of Microstructure Physics, Weinberg 2, 06120 Halle, Germany

[c] Jeong Ha Hwang, Dr. Lucía-Palomino, Prof. Dr. Mickael L. Perrin and Dr. Gabriela Borin Barin

Empa - Swiss Federal Laboratories for Materials Science and Technology

Überlandstrasse 129, 8600 Dübendorf (Switzerland)

E-mail: [Gabriela.BorinBarin@empa.ch](mailto:Gabriela.BorinBarin@empa.ch)

[d] Jeong Ha Hwang, Prof. Dr. Mickael L. Perrin

Department of Information Technology and Electrical Engineering, ETH Zurich, 8092 Zurich, Switzerland

[e] Dr. Ji Ma, College of Materials Science and Optoelectronic Technology &Center of Materials Science and Optoelectronics Engineering,University of Chinese Academy of Science, 100049 Beijing, P. R.China

E-mail: [maji@ucas.ac.cn](mailto:maji@ucas.ac.cn)

[f] Departamento de Química Orgánica, Facultad de Ciencias, Universidad de Granada, Unidad de Excelencia en Química (UEQ), 18071 Granada, Spain

[g] Dr. Sofia Canola

Institute of Physics of the Czech Academy of Science, CZ-16253 Praha, Czech Republic

[h] Dr. José M. Gallego

Instituto de Ciencia de Materiales de Madrid (ICMM), CSIC, Cantoblanco, 28049 Madrid, Spain

[i] Quantum Center, ETH Zürich, 8093 Zürich, Switzerland

[j] Prof. David Écija and Dr. José I. Urgel

Unidad de Nanomateriales avanzados, Imdea Nanoscience, Unidad asociada al CSIC por el ICMM, 28049 Madrid, Spain

[k] Prof. Xinliang Feng

Center for Advancing Electronics Dresden (cfaed) & Faculty of Chemistry and Food Chemistry, Technische Universität Dresden, D-01069 Dresden, Germany

[+] These authors contributed equally to this work.

Supporting information and the ORCID identification number(s) for the author(s) of this article can be found under:

https://doi.org

2020 The Authors. Published by Wiley-VCH Verlag GmbH&Co. KGaA. This is an open access article under the terms of the Creative Commons Attribution-Non Commercial License, which permits use, distribution and reproduction in any medium, provided the original work is properly cited and is not used for commercial purposes

**Table of Contents**

[1. Experimental and computational methods.](#_Toc173398875)

[2. Synthetic details and characterization.](#_Toc173398873)

1. Experimental and computational methods.

The experimental set-up found at IMDEA Nanociencia Institute holds a low-temperature Scienta Omicron STM/nc-AFM housed in a custom-designed UHV system, with a base pressure inside the equipment in the range of low 10^-10^ mbar. d*I*/d*V* spectra and maps were obtained using the lock-in technique (SR830)

The preparation of monocrystal Au(111) surface involved repeated cycles of Ar⁺ ion sputtering at 1.5 keV (11 µA) for 10 minutes, followed by post-annealing at 450 °C for an additional 10 minutes to ensure a clean and well-ordered surface. Precursor **1** was deposited via thermal evaporation (using a Kentax TCE-BSC evaporator) at a sublimation temperature of 200 ºC onto the pre-cleaned Au(111).

For STM imaging, electrochemically etched tungsten tips were used, while nc-AFM images were obtained with a Qplus tuning fork sensor (Scienta Omicron) operating at 4.3 K. The sensor was driven at its resonance frequency of 26 kHz with a constant oscillation amplitude of approximately 60 pm using a MFLI from Zurich Instruments.

**Light experiment**

After deposition at room temperature of precursor **1**, the sample was illuminated with a Super High-Power LED Collimator Sources with a clear aperture of 22-mm (LCS-0470-50-22). The LED source from Mightex was placed on a window from the preparation chamber at 210 mm from the sample. The window is from colloidal glass with a thickness of 3 mm (VAb-Vakuum SFK 40LA). The estimated percentage of light emitted by the LED that reaches the sample is around 2 % from geometric considerations. The LED was driven using 8 A for 15 hours of exposure.

**Raman spectroscopy**

After preparation and characterization of the high-coverage sample in the 10^-7^ mbar range (Figure S8a), the sample was transferred to a vacuum suitcase with an optical access to the Raman microscope. The maximum pressure the sample was exposed to during the transfer and the measurement was in the order of 10^-7^ mbar. Raman spectra were measured in backscattering geometry using Witec Alpha 300 R confocal Raman microscope. The measurements were conducted using a 532 nm excitation at 30 mW and a 600 g/mm grating. Each spectrum was integrated for 15 seconds and recorded at 225 points over a 20 µm ⨉ 20 µm area, then averaged to enhance the S/N ratio. In order to maximize signal intensity, spectra were collected using a 50x LD objective (Zeiss, NA = 0.55), through a 0.2 nm-thick silica window covering a hole of 7 nm in diameter.

**Raman simulation**

The Raman spectrum of a SW polymer composed of 4 monomers was calculated with the ORCA 5.0 DFT code, using PBE exchange-correlation functional, def2-SVP basis set in combination with the RI-J approximation for Coulomb integrals, using the ETH supercomputer (EULER). The frequencies are calculated numerically. Geometry of a structure was relaxed using the same settings.

**DFT-based structural optimization**

For the optimization of the absorption structure of the systems on the Au111 surface we used PBE functional and the FHI-AIMS code. For the infinite polymer, we described the underlaying metallic substrate with a three-layered Au(111) surface with a 6x4 supercell, with a lattice constant of $a=4.0782Å$, periodic boundary conditions and 20 Å of vacuum in the $\hat{z}$ direction, the monomers of precursor **1** on a two-layered Au(111) 8x8 supercell and the seven units finite chain on a one-layered Au(111) 6x36 supercell. During the optimization, the coordinates of the bottom Au layer were kept fix, while the remaining topmost layers were allowed to relax.

**SPM simulations**

The AFM and dI/dV simulated images were obtained by means of the PP-STM code, in the CO functionalized tip modality implemented in the code. The electrostatic potential and eigenstates necessary for the AFM and dI/dV simulations were obtained from DFT simulations of the system using the FHI-AIMS code. The AFM images were obtained using an infinite chain deposited on a 3-layered Au(111) substrate such as the described above, while the dI/dV simulations were performed on a finite polymer, of sever units and terminated by ${CBr}_{2}$. The geometry of the finite polymer was first optimized on a one-layered Au(111) substrate and then, using the obtained coordinates, one self-consistency cycle was performed in gas phase to obtain the eigenstates.

**Absorption spectrum simulation**

The most stable structure of precursor **1** resulting from the DFT relaxation on the surface (“up”) was considered (without surface, in vacuo) and its vertical excited states (at fixed geometry) have been computed with TD-DFT, employing wB97XD functional^[1]^ and 6-31+G* basis set. The absorption spectrum is plotted as “sticks” (excitation energy vs. oscillator strength which indicates the transition intensity), that is dressed with a Lorentzian line broadening (full width half maximum 0.05 eV).

**
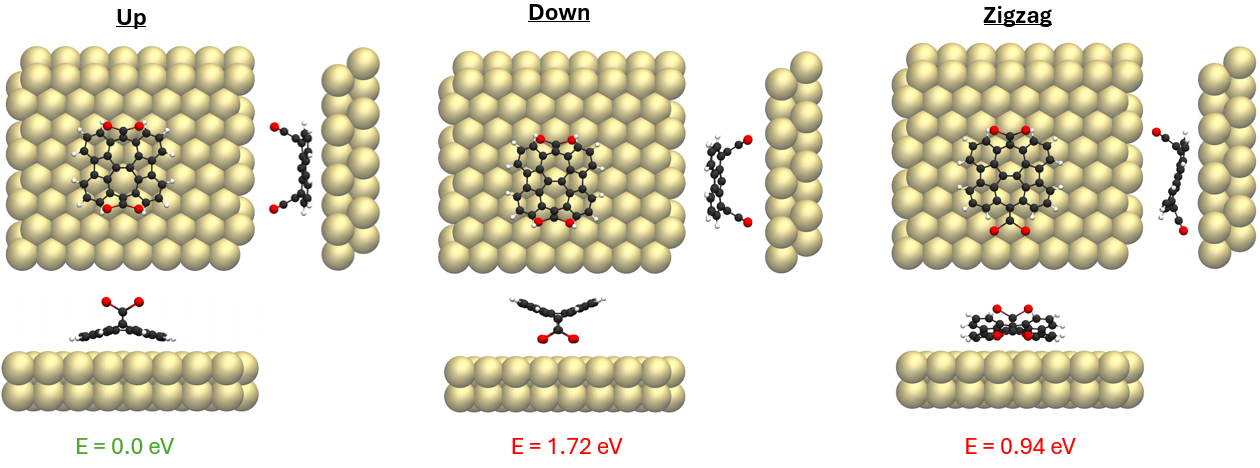
**For comparison, the geometry of **1** has been also optimized in vacuo with PBE functional, 6-31+G* basis set and D3 empirical dispersions.^[2]^ The associated absorption spectrum has been similarly computed with TDDFT, using again wB97XD/6-31+G* level of theory. All calculations were run with Gaussian16 Rev. C.01.^[3]^

**Figure S1.** Computed geometry and total energy of the three considered absorption configurations of the molecular precursor **1** on a two-layered Au(111) surface.

**
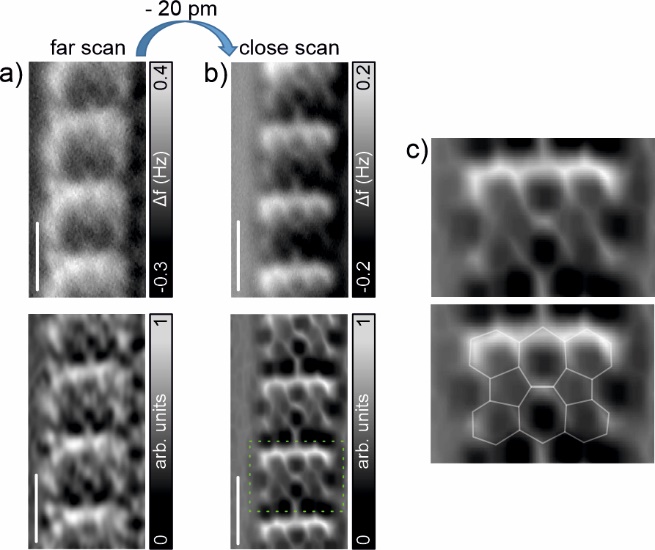
**

**Figure S2. nc-AFM images of the cumulene-linked Stone-Wales polymer segment acquired at different tip heights. a,b)** Height-dependent experimental nc-AFM images of the polymers observed after annealing the surface at 200 ºC. In both cases the absence of bright protrusions at the connection between SW units points toward the cumulene-like nature of the linkage. Constant-height frequency-shift nc-AFM images shown in panels (a) and (b) were acquired using a CO-functionalized tip, at z-offsets of 130 pm and 110 pm, respectively, below the STM set point (5 mV, 50 pA). The corresponding nc-AFM images shown below are processed using Laplace filtering. All scale bars = 1 Å. c) At close tip-polymer distances (i.e. more negative Δz values) the SW defect surrounded by four benzene rings are noticeable, especially in the Laplace filtered images.


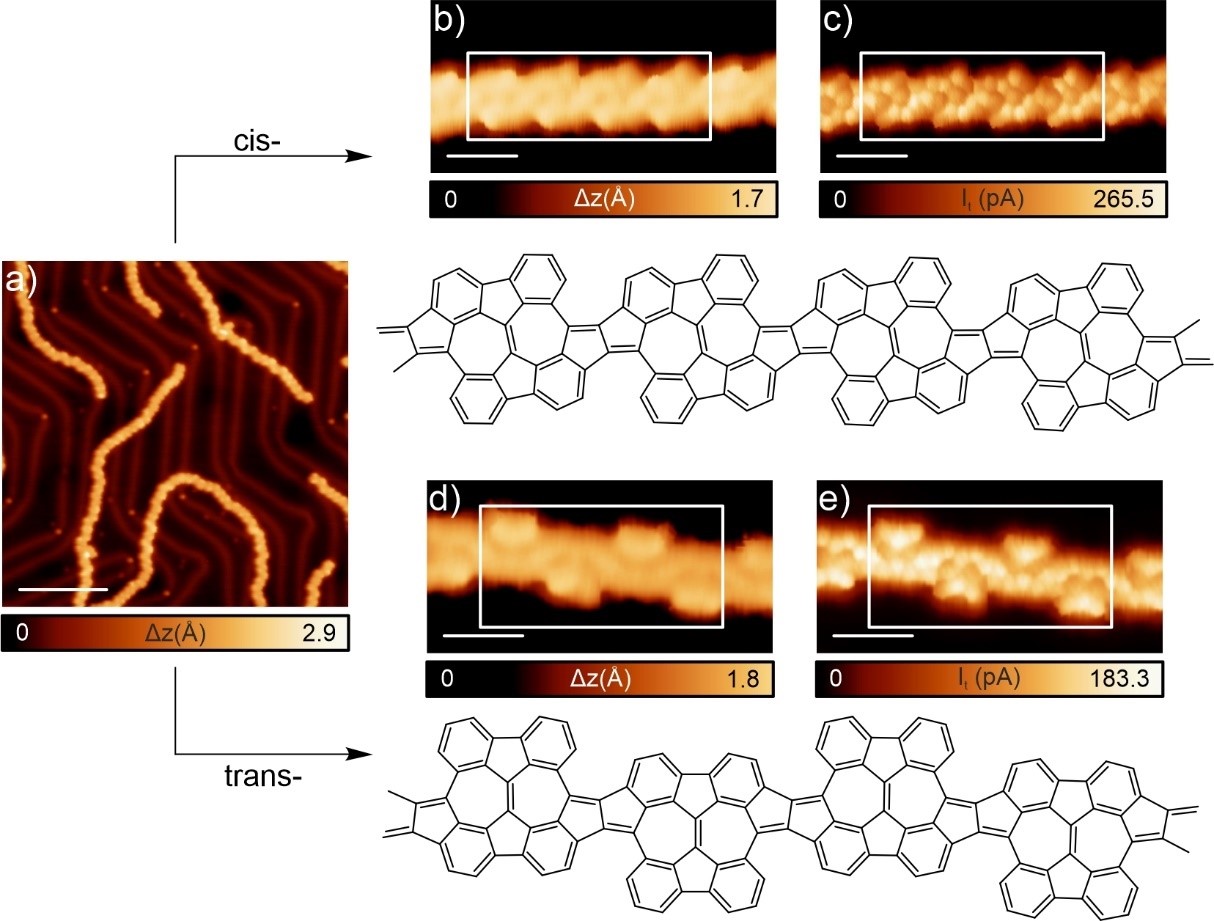


**Figure S3. Ladder SW-based polymers after annealing the sample at 250 ºC.** a) Large-scale high-resolution STM image of irregular ladder SW-based polymers fabricated on Au(111). b-c) Constant-current and constant-height high-resolution STM images of a cis- segment and the corresponding chemical sketch. d-e) Constant-current and constant-height high-resolution STM images of a trans- segment and the corresponding chemical sketch. Scanning parameters: a) V_b_ = −1 V, I_t_ = 50 pA, scale bar = 10 nm, b-e) V_b_ = 5 mV, I_t_ = 50 pA, scale bar = 1nm.

**
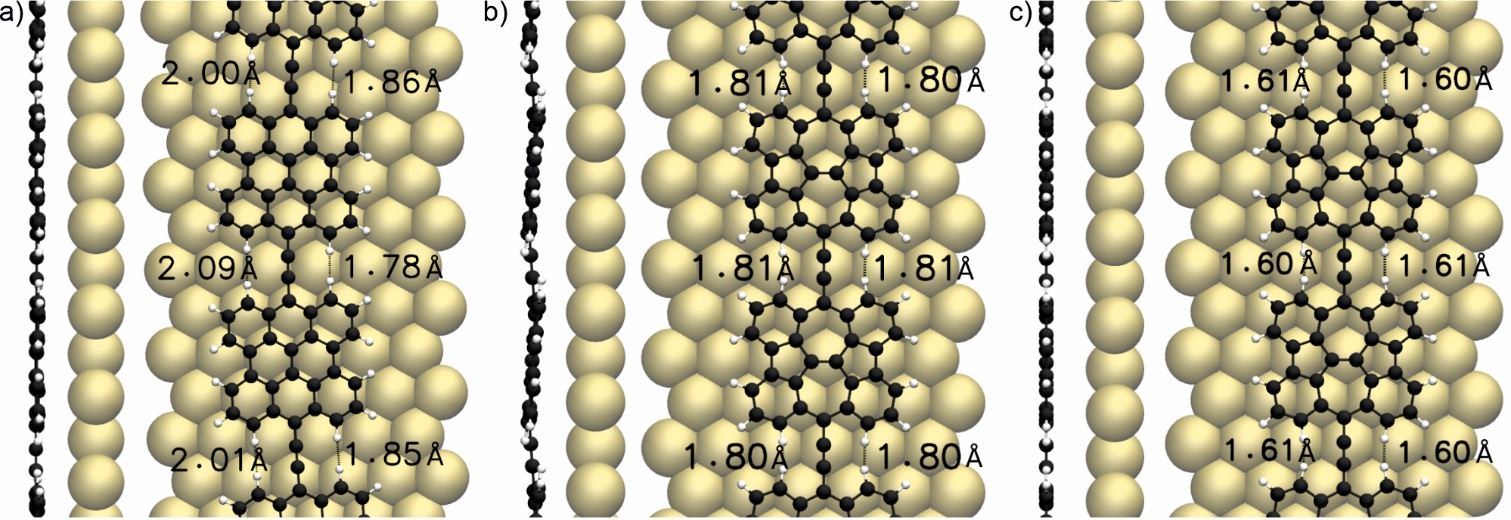
**

**Figure S4.** Absorption structure and H···H distance of optimized a) bisanthene and b) Stone-Wales based polymers and an c) hypothetical completely planar Stone-Wales based polymer. When oligomers of two or more units are formed, the steric hindrance between the hydrogen atoms of neighboring molecules prevents the molecular units from adopting a completely planar configuration. By adopting the observed tilted configuration, the H···H distance in the Stone-Wales polymer was found to be 1.80 Å (See Fig. S3b). This distance is within the range of [1.78, 2.09] Å that we could find for a planar bisanthene polymer, shown in Fig S3a for comparison, and larger than the 1.60 Å found for the Stone-Wales polymer if we “force” a completely planar configuration.


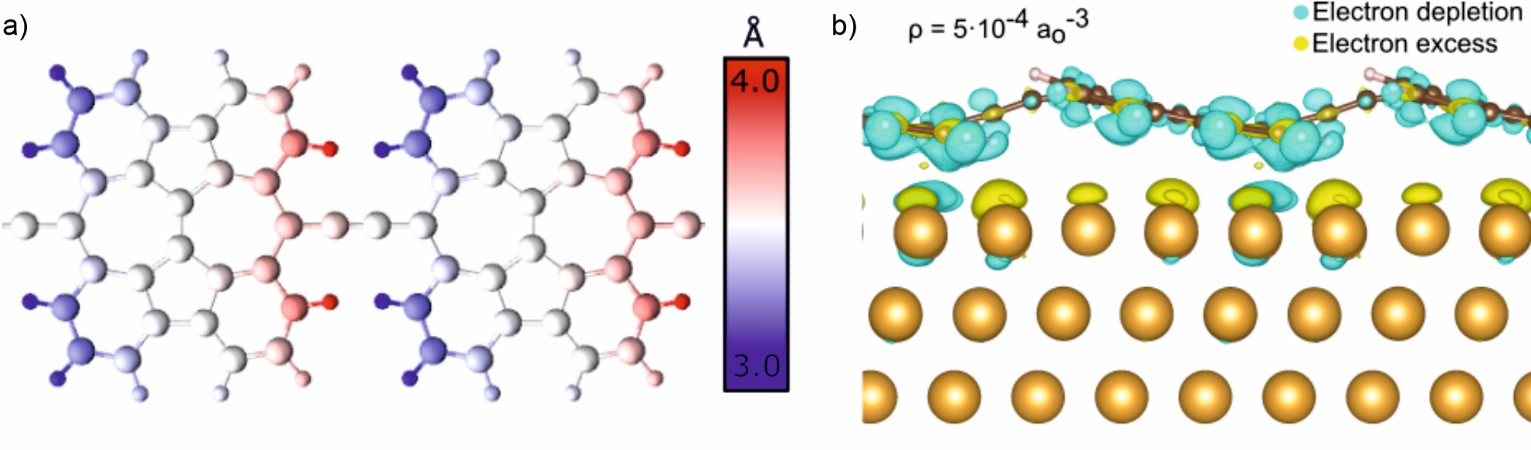


**Figure S5. Absorption height of the polymer with respect to the Au111 surface and charge reorganization upon absorption.** a) Top view of the polymer in which the color of the atoms is given by their height with respect to the underlaying substrate. b) charge density redistribution consequence of the absorption of an infinite chain on an Au(111) three-layered substrate, calculated subtracting from the charge density of the chain deposited on the surface, the charge densities of the chain and sample separately.


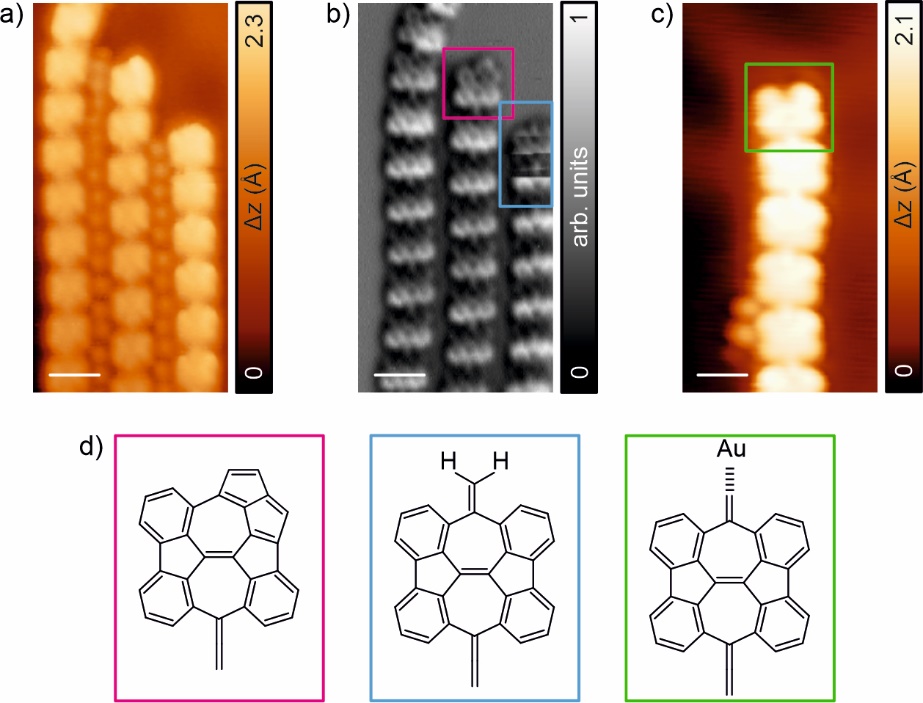


**Figure S6. Irregular terminations and tentative structure identification.** a-b) Constant-current STM image and constant-height frequency shift nc-AFM image of three SW-polymers elucidating two different terminations. c) Constant-current high-resolution STM image of a different SW-polymer termination showing a “bite” at the middle of the edge attributed to the linkage of the terminal carbon atom with the gold surface.^[4]^ d) Corresponding tentative chemical sketches for all three terminations observed, highlighted in pink, blue and green in the experimental images**.** Scanning parameters: a) V_b_ = 5 mV, I_t_ = 50 pA b) V_b_ = 5 mV, I_t_ = 50 pA c) V_b_ = 0.5 V, I_t_ = 50 pA, scale bars: 1 nm.


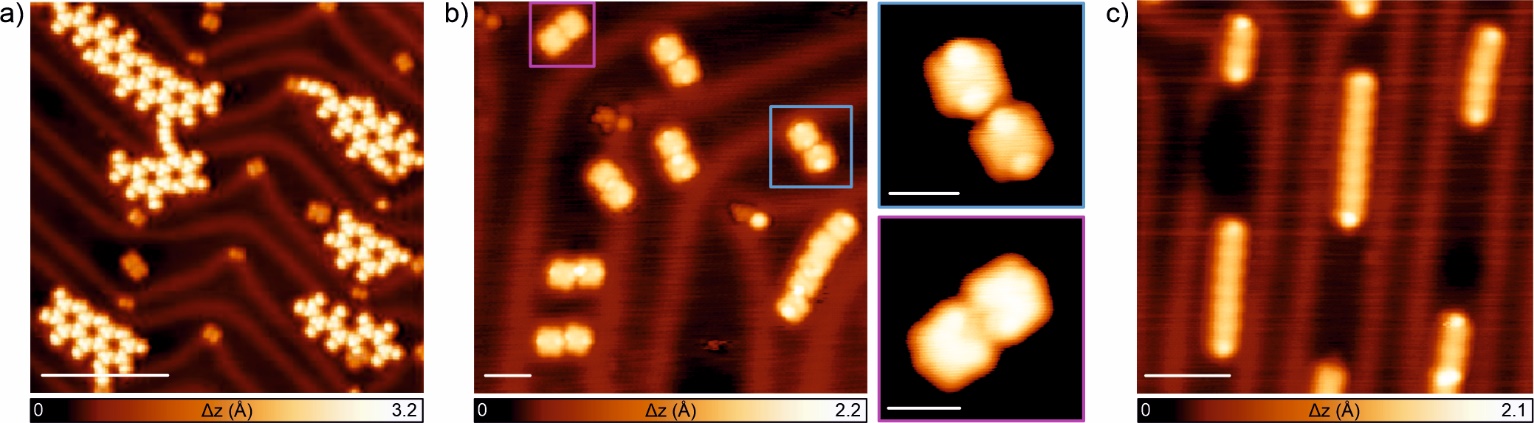
**Figure S7. Exploring the formation of the SW-based polymers after 15 hours: at RT, annealing and illumination.** High-resolution STM images of low coverage samples on the Au(111) surface. a) Deposition of precursor **1** at RT revealing self-assembled molecules and very few oligomers after 15 hours at RT. b) STM image showing the sample containing **1** sublimed at RT and subsequently annealed at 80 ºC for 15 hours. The blue inset unveils the presence of a majority of non-reacted molecules, coexisting with a few polymers and some molecules with the terminal carbon atom linked to the gold surface (pink inset and Figure S3c). c) Light-induced polymerization after exposing the sample for 15 hours with λ= 470 nm LED (see also Figure 2). Sample temperature reaches 80 ºC after the first hour of light exposure. Then it keeps constant till the light irradiation is stopped. Scanning parameters: a) V_b_ = -1 V, I_t_ = 30 pA scale bar: 10 nm, b) V_b_ = 0.5 V, I_t_ = 50 pA, scale bar: 2 nm, c) V_b_ = -2 V, I_t_ = 50 pA, scale bar: 5 nm.

**Molecular absorption spectrum**


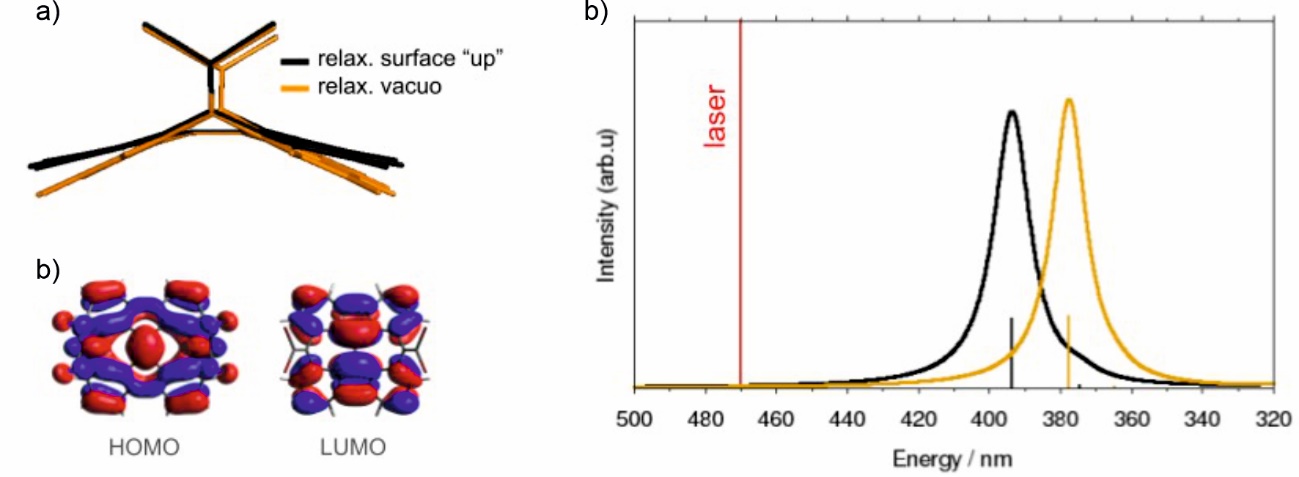


**Figure S8.** (a) Molecular models of **1**: relaxed on the surface (“up” structure, black), relaxed in vacuo (orange). The most significant difference is the bending angle between the fluorene moieties that is significantly reduced on the surface. (b) Computed absorption spectrum of **1** in the low energy spectral window, employing the molecular structures in (a) with the same colour code (without surface) and experimental laser (red line). The peaks energy of the orange structure has a significant red shift, consequence of structural modifications impressed by the adsorption on metal structure: the molecule is flatter and this contributes to the red shift of the main peak by increasing the conjugation. (c) The intense S_1_ excited state dominantly involves a transition between HOMO and LUMO molecular orbitals.

**Table S1.** First intense absorption transition computed with TDDFT at the relaxed structure in vacuo and on surface: excitation energy (in eV and nm) and intensity (oscillator strength).

|  |  | Energy / eV | Energy / nm | Osc.s |  |
| --- | --- | --- | --- | --- | --- |
| Relax vacuo | S_1_ | 3.25 | 378 | 0.313 |  |
| Relax surface “up” | S_1_ | 3.15 | 394 | 0.300 |  |

#
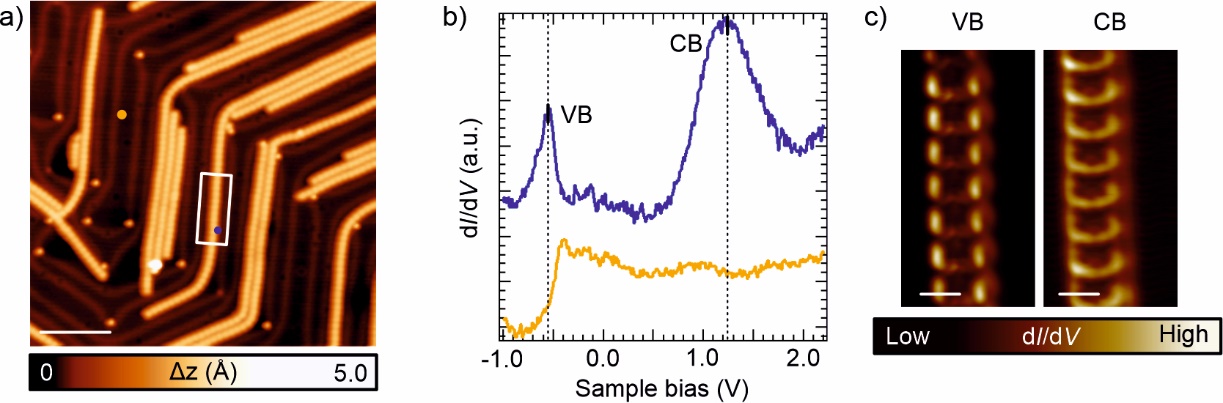


**Figure S9. Electronic structure of an long individual SW-based polymer (> 50 units) on Au(111) .** a) High-resolution STM image acquired with a CO-functionalized tip showing relatively long SW-based polymers. b) d*I*/d*V* spectra acquired at the positions depicted as dots in (a) marked with orange for the bare Au(111) and blue for the SW-based polymer. c) Constant-current differential conductance d*I*/d*V* maps acquired with a CO-tip at the corresponding energetic positions to the VB (left) and the CB (right). Scanning parameters: a) V_b_ = 1 V, I_t_ = 50 pA, scale bar: 10 nm. b) Open feedback parameters for d*I*/d*V* spectra: V_b_ = −1.5 V, I_t_ = 250 pA, V_rms_ = 10 mV. c) V_b_ = −550 mV (VB), V_b_ = 1250 mV (CB), I_t_ = 250 pA, scale bars: 1 nm.


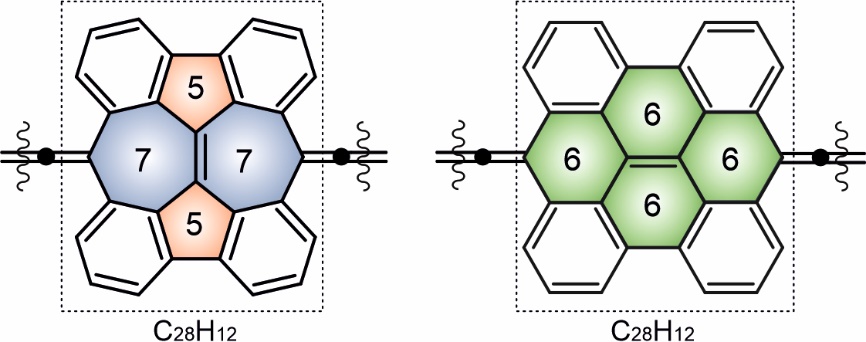


**Figure S10. Structural comparison between cumulene-linked SW- and bisanthene-based polymers.** The chemical structures show that both polymers are isostructural.


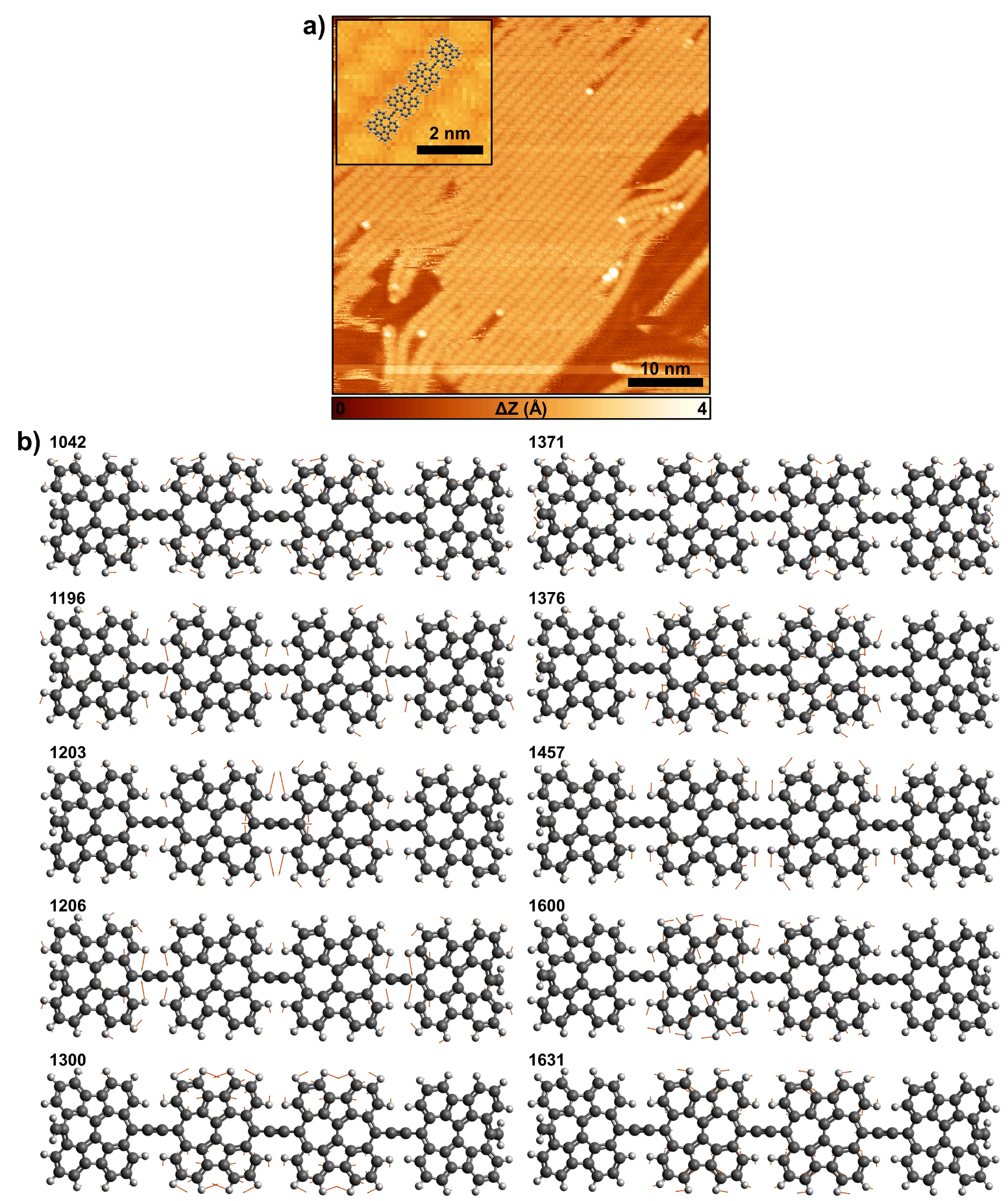
Mode analysis of 4-unit polymer.

**Figure S11. Mode analysis of 4-unit polymer.** a) Large-scale STM image of a high-coverage sample of the SW-based polymers on Au(111)/mica acquired at room temperature. b) Raman normal mode analysis of the 4-unit polymer in the frequencies ranging from ~1040 to 1630 cm^-1^. Red arrows are the force vectors of the atomic displacements.

2. Synthetic details and characterization.

**General Methods and Materials**

Unless otherwise stated, commercially available starting materials, reagents, catalysts, and dry solvents were used without further purification. All reactions were carried out under an inert atmosphere using standard vacuum-line and Schlenk techniques. The starting materials were sourced from TCI, Sigma-Aldrich, abcr, Alfa Aesar, Acros Organics, or Fluorochem, while catalysts were purchased from Strem. Column chromatography was performed on silica gel (SiO₂, particle size 0.063–0.200 mm, VWR), and thin-layer chromatography (TLC) was conducted using silica-coated aluminum sheets with a fluorescence indicator (TLC silica gel 60 F254, Merck KGaA). Deuterated solvents, including dichloromethane-d_2_ (99.9 atom % D) and 1,1,2,2-tetrachloroethane-d_2_ (≥99.5 atom % D), were obtained from Sigma-Aldrich. Recycling gel permeation chromatography (r-GPC) was performed using a JAI HPLC LC 9110 II NEXT system with a fraction collector (FC-3310) and serially connected GPC columns (JAIGEL-1HH, JAIGEL-2HH, JAIGEL-2.5HH), using HPLC-grade chloroform as the eluent.

NMR spectra were recorded on a Bruker AV-II 300 spectrometer operating at 300 MHz for ^1^H and 75 MHz for ^13^C. Unless otherwise specified, measurements were performed at room temperature (296 K). Chemical shifts (δ) are reported in ppm, with coupling constants (*J*) given in Hz. Dichloromethane-d₂ (δ(¹H) = 5.32 ppm, δ(^13^C) = 53.8 ppm) and 1,1,2,2-tetrachloroethane-d_2_ were used as solvents. Peak multiplicities are indicated as follows: s = singlet, d = doublet, t = triplet, q = quartet, and m = multiplet.

Matrix-assisted laser desorption/ionization time-of-flight (MALDI-TOF) MS was recorded on a Bruker Autoflex Speed MALDI-TOF MS (Bruker Daltonics, Bremen, Germany). All of the samples, were prepared by mixing the analyte and the matrix, 1,8-dihydroxyanthracen-9(10H)-one (dithranol, purchased from Fluka Analytical, purity > 98%) or *trans*-2-[3-(4-tert-butylphenyl)-2-methyl-2-propenylidene]malononitrile (DCTB, purchased from Sigma Aldrich, purity > 99%) in the solid state.

**Detailed Synthetic Procedures**

**Synthesis of 9-fluorene-1-carboxylic acid (3)**

The 9-fluorenone-1-carboxylic acid (**3**) is commercially available from TCI Deutschland GmbH, but its high cost makes large-scale use impractical. Fortunately, it can be easily synthesized in large quantities by the oxidation of commercially available fluoranthene (**2**) using chromium trioxide (CrO_3_), following a previously reported procedure.^[5]^

**Synthesis of Methyl 9-fluorene-1-carboxylate (4)**

The florenone derivative **4** was prepared by the reported procedure.^[6,7]^ 9-Fluorenone-1-carboxylic acid (5.0 g, 22.30 mmol) was placed in a 250 mL round-bottom flask equipped with a stir bar and dissolved in methanol (100 mL). Concentrated sulfuric acid (5 mL) was then added, and the reaction mixture was heated to reflux at 80 °C in an oil bath for 20 h. After completion, the solution was cooled and concentrated under vacuum, yielding a yellow powder. The crude product was further purified by column chromatography on silica gel (iso-hexane/ethyl acetate, 4:1) to afford methyl 9-fluorenone-1-carboxylate (**4**) as a yellow powder (4.70 g, 88.5%). ^1^H NMR (300 MHz, CD_2_Cl_2_): δ 7.70-7.30 (m, 7H), 4.00 (s, 3H).

**Synthesis of** **Methyl-9-(2-tosylhydrazineylidene)-9H-fluorene-1-carboxylate (5)**

The synthesis of compound **5** was carried out following a previously reported procedure.^[8]^ A solution of **4** (0.82 g, 3.71 mmol), concentrated HCl (2 mL), and trimethyl orthoformate (0.97 mL, 8.9 mmol) in 22 mL of dry methanol was heated to reflux. Since the tosylhydrazone intermediate rapidly hydrolyzes back to the ketone in solution, trimethyl orthoformate was added to the reaction mixture to scavenge the water formed during the reaction. Tosylhydrazide (0.69 g, 3.71 mmol) was then added, and the solution was refluxed for an additional 16 h. After cooling to room temperature, the resulting yellow precipitate was collected by filtration, affording compound **5** (1.35 g, 89.6% yield), which was used directly in the next step without further purification.

**Synthesis of *(E)* or *(Z)*-****Dimethyl-[9,9′-bifluorenylidene]-1,1′-dicarboxylate (6)**

The synthesis of compound **6** was carried out following a previously reported procedure.^[8]^ A solution of compound **5** (820.3 mg, 2.02 mmol) and sodium methoxide (163.6 mg, 3.03 mmol) in 24 mL of dry pyridine was heated at 110 °C for 6 min, until the reaction mixture bubbled vigorously and turned brown. The mixture was then diluted with 100 mL of water and quickly extracted with dichloromethane (3 × 50 mL). The combined organic layers were dried over Na_2_SO_4_ and evaporated rapidly under reduced pressure with minimal heating.

The resulting crude product was immediately dissolved in 20 mL of dry dichloromethane, and approximately 20 mg of copper(I) bromide was added. The solution was heated to reflux for 15 min. After solvent removal under reduced pressure, the product was purified by column chromatography on silica gel using 5% ethyl acetate/iso-hexane as the eluent, affording compound **6** as a red powder of inseparable isomers (*E/Z*) (401 mg, 89.4% yield over two steps, A/B = 1.72:1). ^1^H NMR spectrum is similar to that reported in the literature.^[6]^ *Isomer A*: ^1^H NMR (300 MHz, CDCl_3_): δ 7.84 (d, 2H), 7.77 (d, 2H), 7.69 (d, 2H), 7.65 (d, 2H), 7.45 (t, 2H), 7.29-7.23 (m, 2H), 7.13 (t, 2H), 3.23 (s, 6H). *Isomer B*: ^1^H NMR (300 MHz, CDCl_3_): δ 8.49 (d, 2H), 7.80 (d, 2H), 7.72 (d, 2H), 7.50 (d, 2H), 7.39-7.31 (m, 2H), 3.01 (s, 6H).

**Synthesis of Heptaleno[2,1,10,9-*jklm*:4,5,6,7-*j'k'l'm'*]difluorene-7,14-dione (7)**

The synthesis of compound **7** was carried out following a previously reported procedure.^[6,7]^ A 10 mL single-neck round-bottom flask was charged with a mixture of compound 6 (500 mg, 1.13 mmol) and trifluoromethanesulfonic acid (1.5 mL) and heated at 90 °C for 3 h. The reaction mixture was then poured onto crushed ice (as illustrated in the attached Figure). The precipitated solid was collected by filtration and sequentially washed with water (2 × 10 mL), acetone (2 × 10 mL), chloroform (2 × 10 mL), and hexane (2 × 10 mL). The obtained solid was dried under vacuum at 100 °C for 5 h, affording compound 7 as an insoluble green solid (430 mg, quantitative yield). The obtained product was too insoluble to record any NMR spectrum and was used directly in the next step without further purification.

**Synthesis of 7,14-Bis(dibromomethylene)-7,14-dihydroheptaleno[2,1,10,9-*jklm*:4,5,6,7-*j'k'l'm*']difluorenedione (1)**

A 20 mL sealed tube was charged with compound **7** (100 mg, 0.263 mmol), PPh_3_ (552 mg, 2.10 mmol), and CBr_4_ (349 mg, 1.05 mmol) in 5 mL of dry, degassed toluene under an argon atmosphere. The reaction mixture was refluxed for 12 h and then cooled to room temperature. The resulting mixture was filtered through Celite and washed with toluene. The filtrate was collected and concentrated under reduced pressure. The crude residue was purified by flash column chromatography on silica gel using a 2:1 (v/v) iso-hexane/CH_2_Cl_2_ eluent, affording a crude yellow solid (67.3 mg, 37.4% yield). The yellow solid was then dissolved in CHCl₃ and further purified by recycle gel-permeation chromatography (GPC). As shown in Figure S2, the peak marked "P" was confirmed as pure compound **1**. After recrystallization from CHCl_3_, the obtained yellow crystals were used as the precursor for on-surface synthesis. The obtained product was too insoluble to record a good ^13^C NMR spectrum. ^1^H NMR (300 MHz, Tetrachloroethane-*d*_2_) δ 7.79 (d, *J* = 7.1 Hz, 4H), 7.61-7.51 (m, 8H). ^13^C NMR (75 MHz, Tetrachloroethane-*d*_2_) δ 141.7, 138.9, 134.5, 132.3, 129.9, 128.8, 120.6, 119.1, 29.8. HR-MALDI-TOF (matrix: dithranol): C_30_H_12_Br_4_, calculated for [M]^+^: 687.7667, found for [M]^+^: 687.7657. CCDC 2302123 contains the X-ray crystallographic coordinates for **1**. These data can be obtained free of charge from Cambridge Crystallographic Data Centre (CCDC) via http://www.ccdc.cam.ac.uk/data_request/cif.


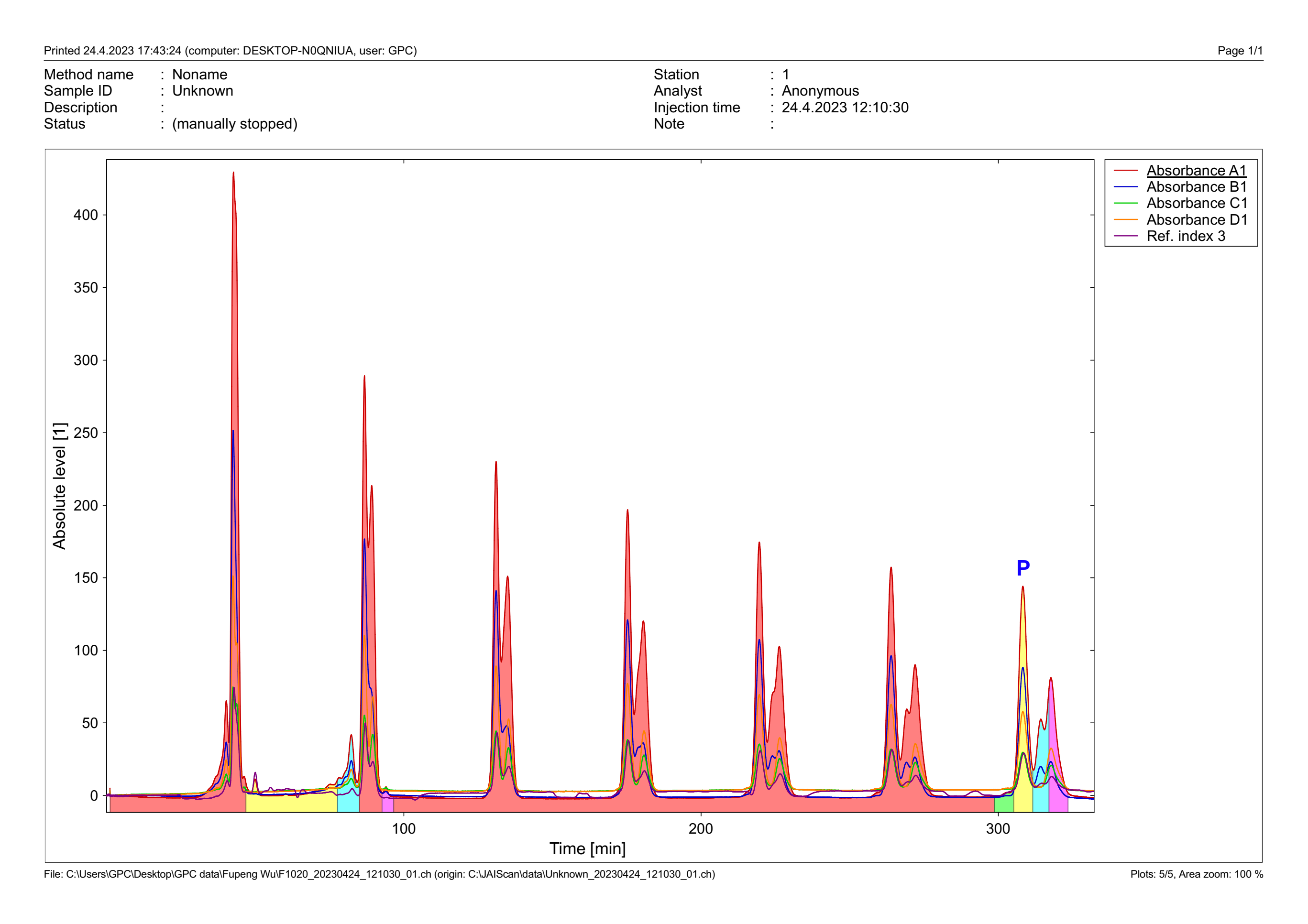


**Figure S12.** Gel-permeation chromatography (GPC) separation spectrum of compound **1**. The fraction from the peak marked "P" was confirmed as pure compound **1**.


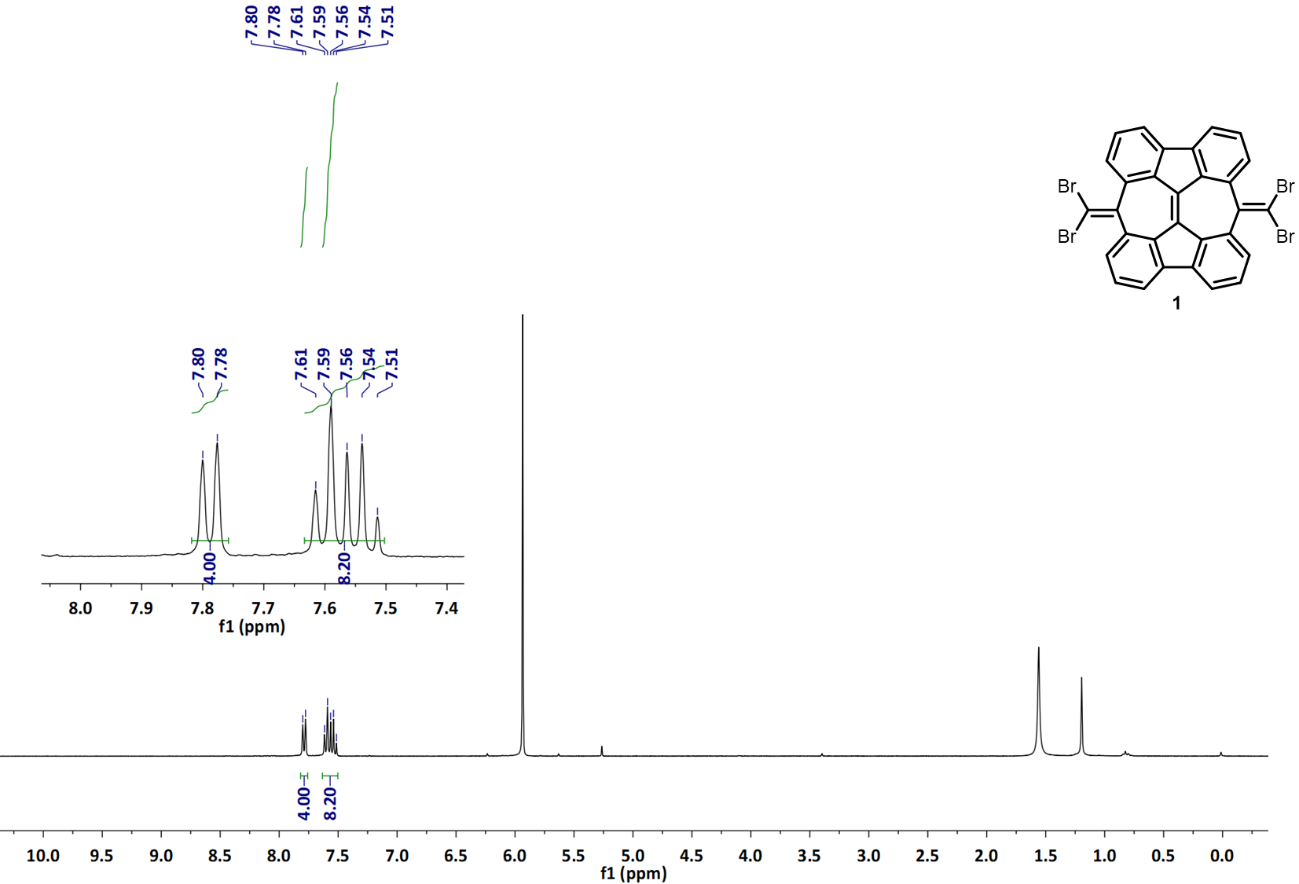


**Figure S13**. ^1^H-NMR spectrum of **1** dissolved in tetrachloroethane-d_2_, 75 MHz, 296 K.


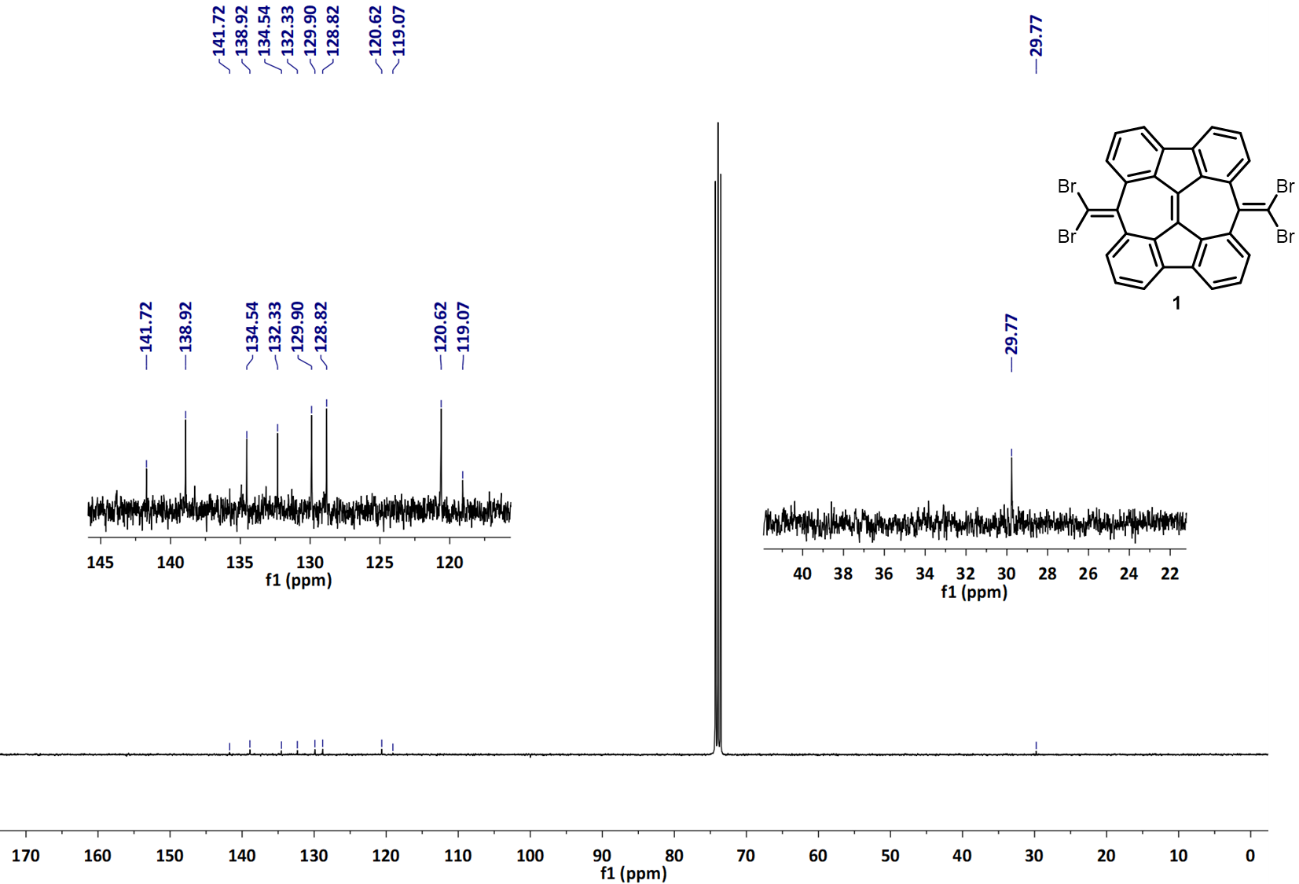


**Figure S14**. ^13^C-NMR spectrum of **1** dissolved in tetrachloroethane-d_2_, 75 MHz, 296 K. The obtained product was too insoluble to record a good ^13^C NMR spectrum.

**X-ray crystallographic analysis**

Single crystal of compound **1** was obtained by the slow diffusion of methanol into the dichloromethane solution. X-ray diffraction data collection was carried out at the BESSY storage ring (BL14.2, Berlin-Adlershof, Germany).^[9]^ XDSAPP2.0 suite was employed for data processing.^[10-11]^ The structure was solved by direct methods and refined by SHELXL-2018.^[12]^ Hydrogen atoms were added geometrically and refined with a riding model.


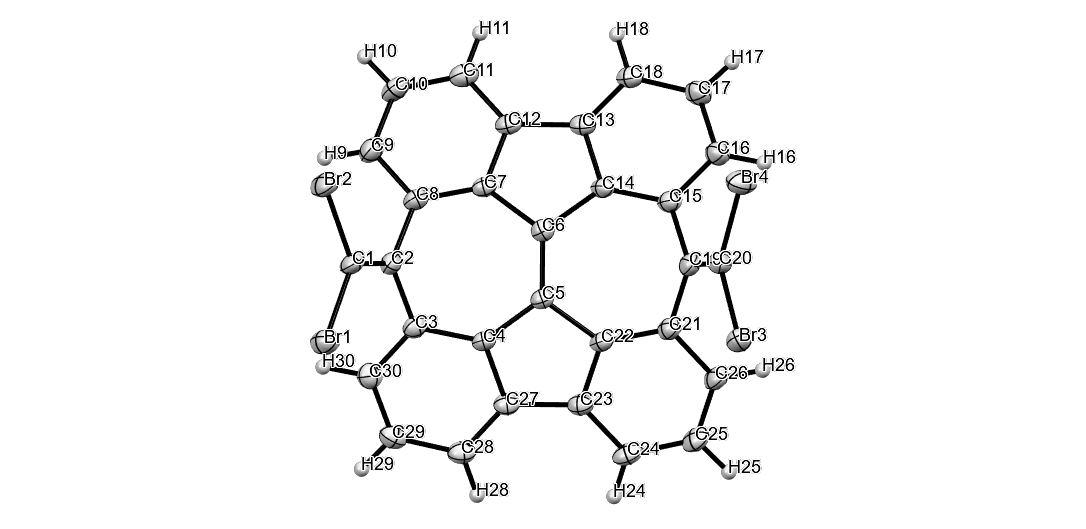


**Figure S15**. X-ray crystallographic structure of **1** (ORTEP drawing with at the 50% probability level).

**Table S2.** Crystallographic data and details of the structure refinements of **1.**

| Crystal | 1 |
| --- | --- |
| Moiety formula | C_30_H_12_Br_4_ |
| Formula weight | 692.04 |
| Crystal size, mm | 0.05x0.05x0.06 |
| Crystal system | triclinic |
| Space group | $P\bar{1}$ |
| *a*, Å | 9.1100(18) |
| *b*, Å | 12.740(3) |
| *c*, Å | 13.000(3) |
| *α*, deg | 113.21(3) |
| *β*, deg | 105.97(3) |
| *γ*, deg | 97.89(3) |
| Volume, Å^3^ | 1280.6(6) |
| *Z* | 2 |
| *D_calcd._, g cm^-3^* | 1.795 |
| *F_000_* | 664 |
| *T*, K | 100 |
| Radiation (λ, Å) | synchrotron (0.7999) |
| *μ*, mm^-1^ | 8.580 |
| 2Θ range (°) | 4.076-73.284 |
| Index ranges | -13 ≤ h ≤ 13, -18 ≤ k ≤ 18, -18 ≤ l ≤ 18 |
| no. of collected reflections | 7043 |
| no. of unique ref. (*R*_int_) | 5977 (0.0807) |
| Data/restraints/parameters | 7043/0/307 |
| *R*_1_, w*R*_2_ [obs *I*>2*σ* (*I*)] | 0.0656, 0.1897 |
| *R*_1_, w*R*_2_ (all data) | 0.0720, 0.1948 |
| residual peak/hole, e. Å^-^*^3^* | 2.177/-2.038 |
| Goodness-of-fit on *F*^2^ | 1.078 |
| CCDC | 2302123 |

References.

[1] J.-D. Chai, M. Head-Gordon, *Phys. Chem. Chem. Phys.* **2008**, *10*, 6615–6620.

[2] S. Grimme, J. Antony, S. Ehrlich, H. Krieg, *J. Chem. Phys.* **2010**, *132*, 154104.

[3] M. J. Frisch, G. W. Trucks, H. B. Schlegel, G. E. Scuseria, M. A. Robb, J. R. Cheeseman, G. Scal-mani, V. Barone, G. A. Petersson, H. Nakatsuji, X. Li, M. Caricato, A. V. Marenich, J. Bloino, B. G. Janesko, R. Gomperts, B. Mennucci, H. P. Hratchian, J. V., *Gaussian, Inc., Wallingford CT* **2016**.

[4] A. Sánchez-Grande, B. de la Torre, J. Santos, B. Cirera, K. Lauwaet, T. Chutora, S. Edalatmanesh, P. Mutombo, J. Rosen, R. Zbořil, R. Miranda, J. Björk, P. Jelínek, N. Martín, D. Écija, *Angew. Chem.* **2019**, *131*, 6631–6635.

[5] S. R. D. George, L. T. Scott, J. B. Harper, *Polycycl. Aromat. Compd.* **2016**, *36*, 697.

[6] A. Konishi, K. Horii, D. Shiomi, K. Sato, Takeji Takui, M. Yasuda, *J. Am. Chem. Soc*. **2019**, *141*, 10165−10170.

[7] X. Aguilar-Enriquez, L. P. Skala, W. R. Dichtel, *J. Org. Chem*. **2022**, *87*, 16307−16312

[8] H. E. Bronstein, N. Choi, L. T. Scott, *J. Am. Chem. Soc*. **2002**, *124*, 8870-8875.

[9] U. Mueller, R. Förster, M. Hellmig, F. U. Huschmann, A. Kastner, P. Malecki, S. Pühringer, M. Röwer, K. Sparta, M. Steffien, M. Ühlein, P. Wilk, M. S. Weiss, *Eur. Phys. J. Plus* **2015,** *130*, 141.

[10] W. Kabsch, *Acta Cryst. D* **2010,** *66*, 125-132.

[11] K. M. Sparta, M. Krug, U. Heinemann, U. Mueller, M. S. Weiss, *J. Appl. Cryst.* **2016,** *49*, 1085-1092.

[12] G. Sheldrick, *Acta Cryst. C* **2015,** *71*, 3-8.
